# The development and external validation of simplified T category classification for nasopharyngeal carcinoma to improve the prognostic value in the intensity‐modulated radiotherapy era

**DOI:** 10.1002/cam4.2131

**Published:** 2019-04-04

**Authors:** Ling‐Long Tang, Shao‐Bo Liang, Cheng‐Long Huang, Fan Zhang, Cheng Xu, Yan‐Ping Mao, Li Tian, Ai‐Hua Lin, Li Li, Ying Sun, Jun Ma

**Affiliations:** ^1^ Department of Radiation Oncology Sun Yat‐sen University Cancer Center, State Key Laboratory of Oncology in South China, Collaborative Innovation Center for Cancer Medicine, Guangdong Key Laboratory of Nasopharyngeal Carcinoma Diagnosis and Therapy Guangzhou People’s Republic of China; ^2^ Department of Radiation oncology Cancer Center, First People’s Hospital of Foshan Affiliated to Sun Yat‐sen University Foshan People’s Republic of China; ^3^ Imaging Diagnosis and Interventional Center State Key Laboratory of Oncology in South China, Sun Yat‐sen University Cancer Center, Collaborative Innovation Center for Cancer Medicine Guangzhou People’s Republic of China; ^4^ Department of Medical Statistics and Epidemiology School of Public Health, SunYat‐sen University Guangzhou People’s Republic of China

**Keywords:** external validation, intensity‐modulated radiotherapy, nasopharyngeal carcinoma, prognosis, T category classification

## Abstract

**Background:**

Intensity‐modulated radiotherapy (IMRT) provides excellent local control in nasopharyngeal carcinoma (NPC). We investigated whether simplifying 8th American Joint Committee on Cancer staging system T categories improves prognostic value.

**Methods:**

We used 2191 NPC patients as a training set and 414 patients separately as an independent, external validation cohort.

**Results:**

In the training set, local relapse‐free survival (LRFS), disease‐free survival (DFS), and overall survival (OS) were not significantly different between the 8th edition T2/T3 (*P = *0.610, 0.380 and 0.353, respectively). Merging T2 and T3 to proposed T2 (proT2) provided significant differences in LRFS, DFS, and OS between proposed T categories. Proposed T categories had similar c‐indices for LRFS, DFS, and OS (vs the 8th edition), which was validated in the external cohorts. Moreover, for DFS, the adjusted HRs of the proT2N0 (3.8), proT1N1 (3.8), and proT2N1 (6.0) subsets were similar; the adjusted HRs of the proT3N0 (7.0), proT3N1 (11.4), proT1N2 (11.0), proT2N2 (11.6), and proT3N2 (13.3) subsets were similar; the adjusted HRs of the proT1N3 (17.8), proT2N3 (15.3), and proT3N3 (26.4) subsets were similar; the results of the adjusted HRs for OS had the same rule. Defining proT1N0 as stage I; proT1N1/proT2N0‐1 as stage II; proT3N0‐2/proT1‐2N2 as stage III; and proT1‐3N3 as stage IVa generated orderly, significant differences in DFS and OS between stages in the training set and external validation cohort.

**Conclusions:**

In the IMRT era, three T categories are more reasonable (merging T2/T3 into T2) and proT3N0‐2 (the 8th edition T4N0‐2) should be down‐staged to stage III.

## 1 INTRODUCTION

Nasopharyngeal carcinoma (NPC) arises from the nasopharyngeal epithelium and has an extremely unbalanced geographical distribution, with a high age‐standardized incidence of 20‐50 per 100 000 males in southern China.^1^

An accurate TNM staging system is crucial for not only predicting prognosis but also guiding clinicians when making treatment decisions for different risk groups and evaluating treatment outcomes between centers. The TNM staging system for NPC has been modified several times to reflect new developments in diagnostic and therapeutic techniques. Recently, the American Joint Committee on Cancer/International Union against Cancer (AJCC/UICC) released the 8th edition of the TNM staging system to further help physicians assign the appropriate treatments and evaluate treatment outcomes and clinical trials.^2^

The 8th edition made some revisions based on the 7th edition, including changing medial and lateral pterygoid muscle involvement from T4 to T2, adding prevertebral muscle involvement as T2, replacing the supraclavicular fossa with the lower neck, merging N3a and N3b to create N3, and merging T4 and N3 to create stage IVa. The 8th edition of the AJCC NPC staging system has been proven to provide more accurate prediction of treatment outcomes than the 7th edition.^2^

Due to anatomic constraints and its high radiosensitivity, radiotherapy is the primary and only curative treatment for nonmetastatic NPC. Intensity‐modulated radiation therapy (IMRT) was a pioneering breakthrough that significantly improved outcomes; the local control rate for NPC is currently 90%‐95% for patients treated using modern techniques.^3^, ^4^, ^5^ However, these advances have altered the prognostic value of staging parameters for local failure,^6^ and the prognostic value of T category may have become weaker. Indeed, our previous study showed the survival curves for T2 and T3 almost overlapped, with no significant differences in locoregional relapse‐free survival and disease‐free survival (DFS).^2^

Thus, due to the improved local control provided by modern techniques, we reevaluated the prognostic value of the 8th edition T categories by analyzing a large cohort of patients treated with IMRT in this study, with the aim of proposing improvements for the next edition of the AJCC/UICC staging system for NPC.

## 2 MATERIALS AND METHODS

### 2.1 Patient characteristics

All 2605 patients with newly diagnosed, nondistant metastatic, and histologically proven NPC treated with IMRT were retrospectively reviewed. All patients completed a pretreatment evaluation, including complete patient history, physical examination, hematology and biochemistry profiles, neck and nasopharyngeal magnetic resonance imaging (MRI), chest radiography, abdominal sonography, and single‐photon emission computed tomography whole body bone scan or (18)F‐fluorodeoxyglucose (18F‐FDG) positron emission tomography CT (PET/CT) examination. All patients were restaged according to the 8th edition staging system. A total of 2191 patients were recruited at Sun Yat‐Sen University Cancer Center between November 2009 and October 2012 as a training set, and 414 patients collected from the First People's Hospital of Foshan (Foshan, China) between April 27, 2010 and March 3, 2014. The latter group was separately used as an independent, external validation cohort. The clinicopathological characteristics of the patients are summarized in Table 1. The authenticity of this article has been validated by uploading the key raw data onto the Research Data Deposit public platform (http://www.researchdata.org.cn), with the approval RDD number as RDDA2019000962.

**Table 1:** Clinicopathological characteristics of the 2191 patients with NPC

Characteristic	Training cohort (n = 2191)	External cohort (n = 414)
Sex		
Male	1639 (74.8%)	313 (75.5%)
Female	552 (25.2%)	101 (24.2%)
Age (years)		
≤50	1492 (68.1%)	236 (57.0%)
>50	699 (31.9%)	178 (435)
Histological type		
Keratinizing squamous cell carcinoma	12 (0.5%)	0
Nonkeratinizing carcinoma	2179 (99.5%)	414 (100%)
Chemotherapy		
Yes	1900 (86.7%)	349 (84.3%)
No	291 (13.3%)	65 (15.7%)
Induction chemotherapy	1106(50.5%)	228 (55.1%)
Concurrent chemotherapy	1631(74.4%)	317 (76.6%)
Adjuvant chemotherapy	70(3.2%)	1 (0.2%)
T category		
T1	379 (17.3%)	118 (28.5%)
T2	361 (16.5%)	70 (16.9%)
T3	1036 (47.3%)	130 (31.4%)
T4	415 (18.9%)	96 (23.2%)
N category		
N0	363 (16.6%)	54 (13.0%)
N1	1219 (55.6%)	227 (54.8%)
N2	313 (14.3%)	98 (23.7%)
N3	296 (13.5%)	35 (8.5%)
Stage		
I	128 (5.8%)	27 (6.5%)
II	433(19.8%)	109 (26.3%)
III	970 (44.3%)	151 (36.5%)
IV	660 (30.1%)	127 (30.7%)

Note

According to the 8th edition of the AJCC NPC staging system. NPC, nasopharyngeal carcinoma.

### 2.2 Treatment

The nasopharyngeal and neck tumor volumes of all patients were treated using radical radiotherapy based on IMRT for the entire course. Target volumes were delineated slice‐by‐slice on treatment planning CT scans using an individualized delineation protocol.^7^ The prescribed doses were 66‐72 Gy/28‐33 fractions to the planning target volume (PTV) of the primary gross tumor volume (GTVnx), 64‐70 Gy/28‐33 fractions to the PTV of the GTV of involved lymph nodes (GTVnd), 59.4‐63 Gy/28‐33 fractions to the PTV of the high‐risk clinical target volume (CTV1), and 50.4‐56 Gy/28‐33 fractions to the PTV of the low‐risk clinical target volume (CTV2). All targets were treated simultaneously using the simultaneous integrated boost technique. During the study, institutional guidelines recommended only IMRT for stage I NPC and IMRT combined with concurrent chemoradiotherapy ± neoadjuvant/adjuvant chemotherapy for stage II to IVa NPC. When possible, salvage treatments (intracavitary brachytherapy, surgery, or chemotherapy) were provided for patients with documented relapse or persistent disease.^6^

### 2.3 Follow‐up and endpoints

Patient follow‐up was measured from first day of therapy to last examination or death. Patients were examined at least every 3 months during the first 2 years, then every 6 months for at least 3 years and annually thereafter or until death. Median follow‐up was 62.3 months (range, 1.2‐91.5 months) in the training set and 52.0 months (range, 2.0‐83.0 months).

The following endpoints (time from day 1 of treatment to the date of first defining event) were assessed: DFS, to failure or death from any cause, whichever occurred first; overall survival (OS), to death; distant metastasis‐free survival (DMFS), to first distant failure; LRFS, to first local failure; and nodal relapse‐free survival (NRFS), to first regional failure. Patients with residual or recurrent local disease underwent biopsy to confirm malignancy. Additional tests were ordered when indicated to evaluate for local or distant failure.

### 2.4 Statistical analysis

All analyses were performed using SPSS version 20.0 (IBM Corporation, Armonk, NY). Actuarial rates were estimated using the Kaplan‐Meier method; survival curves were compared using the log‐rank test.^8^ Multivariate analyses using the Cox proportional hazards model were used to test for independent significance by backward elimination of insignificant explanatory variables.^9^ The Cox proportional hazards model was also used to calculate hazard ratios (HR). The performance of the 8th edition of the AJCC/UICC staging system and the proposed staging system were also compared using Harrell's concordance index (c‐index).^10^ The c‐index measures ability to predict outcomes; a higher c‐index suggests a greater ability to discriminate outcomes (ie, the model has better discriminatory power).

Host factors (age and gender) and treatment (chemotherapy) were included as covariates in all tests. N category was included as a covariate in the T category analysis. Two‐tailed *P*‐values <0.05 were considered statistically significant.

## 3 RESULTS

### 3.1 Patterns of treatment failure and survival

A total of 458/2191 (20.9%) patients developed recurrence, distant metastasis, or died: 133 (6.1%) developed local recurrence, 106 (4.8%) developed nodal recurrence, 269 (12.3%) developed distant metastasis, and 308 (14.1%) died. Median time was 27.0 (range, 4.5‐70.0) months to local recurrence; 28.5 (range, 1.2‐70.0) months, to nodal recurrence; and 18.4 (range, 1.8‐74) months, to distant metastasis. The 3‐year OS, DFS, DMFS, LRFS, and NRFS rates for the entire cohort were 92.3%, 84.1%, 89.8%, 95.7%, and 97.0%, respectively. The 5‐year OS, DFS, DMFS, LRFS, and NRFS rates for the entire cohort were 86.3%, 79.9%, 87.8%, 93.4%, and 95.5%, respectively.

### 3.2 Prognostic value of the 8th edition T categories

**Figure 1:**
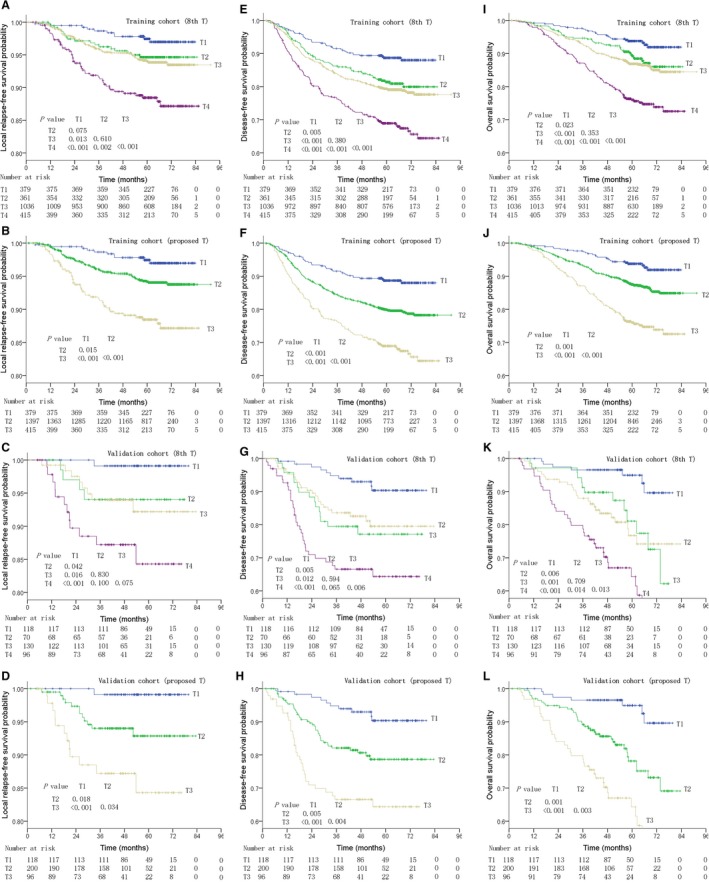
Local relapse‐free survival (A, C; B, D; respectively), disease‐free survival (E, G; F, H; respectively), and overall survival (I, K; J, L; respectively) for different T categories of nasopharyngeal cancer as defined by the 8th edition of the AJCC staging system, and the proposed staging system.

T category of the 8th edition was an independent prognostic factor for local failure and disease failure in multivariate analysis (*P < *0.01). For patients with T1, T2, T3, and T4 NPC, the 5‐year LRFS rates were 97.4%, 94.6%, 94.0%, and 88.4%, respectively; DFS rates were 93.8%, 89.4%, 86.8%, and 76.0%, respectively; and OS rates were 93.8%, 89.4%, 86.9%, and 76.0%, respectively. However, LRFS was not significantly different between patients with T1, T2, and T3 NPC (T1 vs T2, *P = *0.075; T2 vs T3, *P = *0.610). DFS and OS was significantly different between T1 and T2 (*P = *0.005, *P = *0.023, respectively), but not between T2 and T3 (*P = *0.380, *P = *0.353, respectively, Figure 1). Data were adjusted by other potential clinical prognostic covariates including age (>50 years vs ≤50), gender (female vs male), N‐classification, and chemotherapy (yes vs no), and the HRs for LRFS in T1, T2, T3, and T4 NPC were 1.000, 1.882, 1.915, and 3.646, respectively; for DFS were 1.000, 1.320, 1.469, and 2.464, respectively; and for OS were 1.000, 1.285, 1.414, and 2.651, respectively (Table 2). This finding was also validated in the external cohort (Figure 1, Table 2).

**Table 2:** Risks of different T category for LRFS, DFS, and OS based on the 8th edition and proposed staging system for NPC.

Category	Hazard ratio (95% CI) for LRFS		Hazard ratio (95% CI) for DFS		Hazard ratio (95% CI) for OS	
	8th edition	Proposed	8th edition	Proposed	8th edition	Proposed
T category (Training cohort)						
T1	1	1	1	1	1	1
T2	1.882 (0.857‐4.131)	1.905 (0.967‐3.753)	1.320 (0.880‐1.980)	1.426 (1.015‐2.003)	1.285 (0.768‐2.150)	1.379 (0.896‐2.121)
T3	1.915 (0.954‐3.843)	3.643 (1.760‐7.538)	1.469 (1.035‐2.084)	2.453 (1.689‐3.563)	1.414 (0.909‐2.200)	2.644 (1.667‐4.193)
T4	3.646(1.761‐7.552)		2.464 (1.696‐3.582)		2.651 (1.671‐4.206)	
c‐index	0.618 (0.568‐0.668)	0.629 (0.581‐0.677)	0.592 (0.564‐0.620)	0.594 (0.567‐0.621)	0.634 (0.599 −0.669)	0.639 (0.605‐0.674)
T category (Validation cohort)						
T1	1	1	1	1	1	1
T2	7.244 (0.809‐64.845)	7.902 (1.027‐60.8160)	2.208 (0.984‐4.950)	2.127 (1.056‐4.285)	3.367 (1.320‐8.587)	3.731 (1.644‐8.467)
T3	8.279 (1.035‐66.246)	18.322 (2.380‐141.040)	2.080 (0.988‐4.377)	4.894 (2.404‐9.965)	3.952 (1.684‐9.277)	8.117 (3.518‐18.727)
T4	18.323 (2.380‐141.048)		4.893 (2.403‐9.963)		8.152 (3.533‐18.812)	
c‐index	0.722 (0.627‐0.817)	0.723 (0.633‐0.813)	0.708 (0.654‐0.762)	0.723 (0.671‐0.748)	0.708 (0.641‐0.776)	0.706 (0.638‐0.773)

Note

Hazard ratios were calculated using an adjusted Cox proportional hazards model. The following known important prognostic variables were included in the Cox proportionalhazards model: age (>50 years vs ≤50), gender (female vs male), N‐classification, and chemotherapy (yes vs no). NPC: nasopharyngeal carcinoma. LRFS: local relapse‐free survival. DFS: disease‐free survival. OS: overall survival. CI: confidence interval. c‐index: concordance index.

### 3.3 Proposed T category classification

In the 8th edition AJCC staging system, parapharyngeal extension and adjacent soft tissue involvement (medial pterygoid, lateral pterygoid, prevertebral muscles) are classified as T2 disease, and bony structures (skull base, cervical vertebra) and/or paranasal sinuses are classified as T3 disease. Parapharyngeal space, medial pterygoid, lateral pterygoid, prevertebral muscle, bony structure, and paranasal sinus involvement were analyzed in univariate analysis and multivariate analysis of patients with T2‐3 disease. None of these factors had significant prognostic value for LRFS in patients with T2‐3 disease in either univariate or multivariate analysis (Table S1).

Merging T2 and T3 into a proposed T2 (proT2) category seems a reasonable alteration; therefore, the T category classification would contain three categories instead of four. Using this T category reclassification (proT1, proT2, and proT3), the 5‐year LRFS rates were 97.4%, 94.2%, and 88.4%, respectively; the DFS rates were 88.7%, 79.9%, and 68.9%, respectively; and the OS rates were 93.8%, 87.5%, and 76.0%, respectively. Significant differences in LRFS, DFS, and OS were observed between each proposed T category (Figure 1). In proT1, proT2, and proT3 NPC, the adjusted HRs [adjusted by age (>50 years vs ≤50), gender (female vs male), N‐classification, and chemotherapy (yes vs no)] for LRFS were 1.000, 1.905, and 3.643, respectively; for DFS were 1.000, 1.426, and 2.453, respectively; and for OS were 1.000, 1.379, and 2.644, respectively. Compared to the 8th edition, the proposed T category classification had similar c‐indices for LRFS, DFS, and OS; this finding was also validated in the external cohort (Figure 1, Table 2).

### 3.4 Proposed overall stage classification

**Figure 2:**
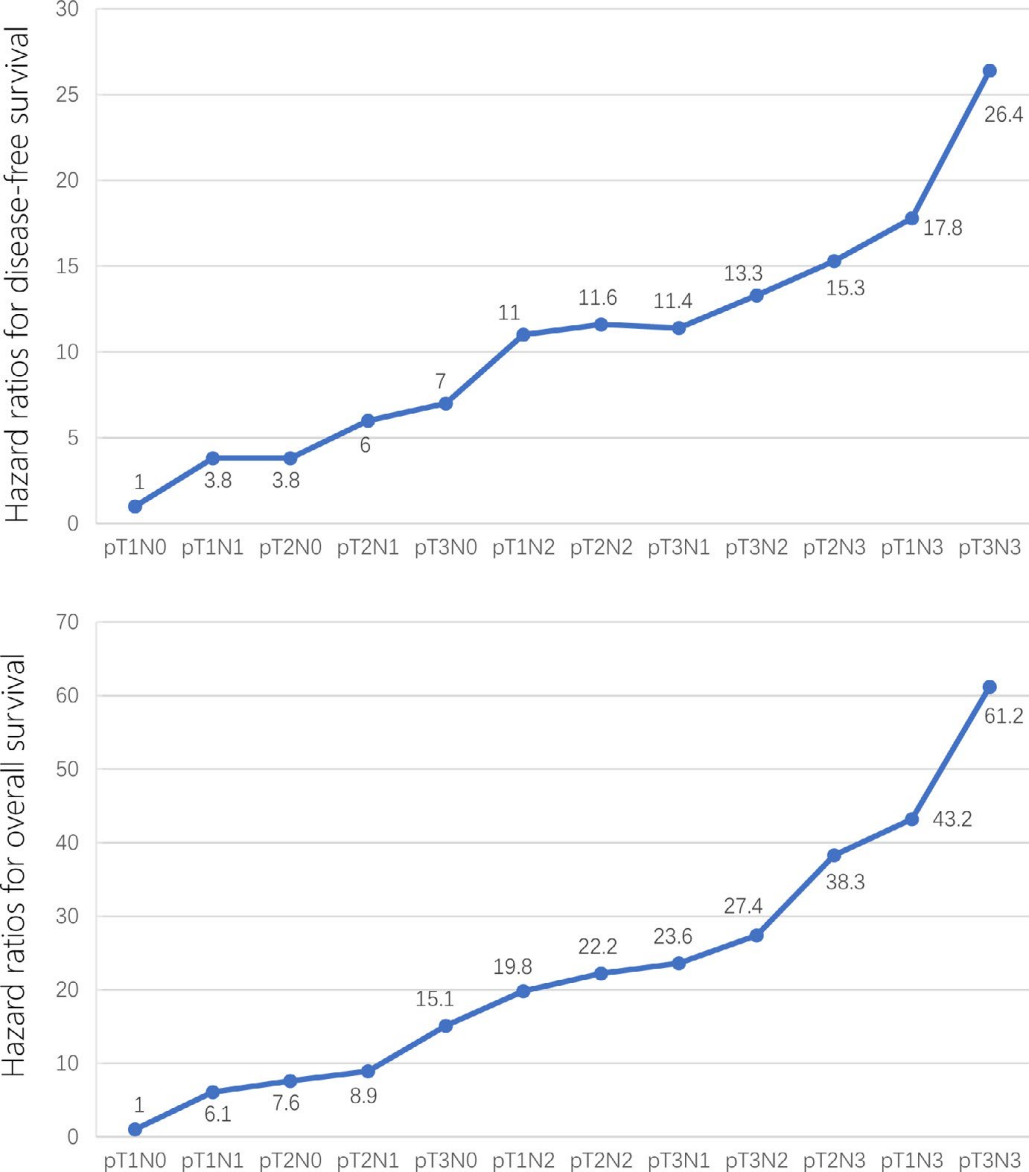
Adjusted hazard ratios (HR) of disease‐free survival and overall survival for different subsets of patients with nasopharyngeal cancer based on the proposed T and N categories

All 2191 patients were classified into 12 groups according to the following proposed T and N categories: proT1N0, proT2N0, proT1N1, proT2N1, proT3N0, proT3N1, proT3N2, proT1N2, proT2N2*,* proT1N3, proT2N3, and proT3N3. The HRs for DFS and OS for each of the 12 subsets were calculated to assess the homogeneity of the prognosis of each T and N subset within each stage. The HR data were generated by Cox regression analysis, with each subset represented by an indicator variable and proT1N0 as the reference group. Interestingly, for DFS, the adjusted HRs [adjusted by age (>50 years vs ≤50), gender (female vs male), and chemotherapy (yes vs no)] of the proT2N0 (3.8), proT1N1 (3.8) and proT2N1 (6.0) subsets were similar; the adjusted HRs of the proT3N0 (7.0), proT3N1 (11.4), proT1N2 (11.0), proT2N2 (11.6) and proT3N2 (13.3) subsets were similar; and the adjusted HRs of the proT1N3 (17.8), proT2N3 (15.3) and proT3N3 (26.4) subsets were similar. For OS, the adjusted HRs of the proT2N0 (7.6), proT1N1 (6.1) and proT2N1 (8.9) subsets were similar; the adjusted HRs of the proT3N0 (15.1), proT3N1 (23.6), proT1N2 (19.8), proT2N2 (22.2) and proT3N2 (27.4) subsets were similar; and the adjusted HRs of the proT1N3 (43.2), proT2N3 (38.3) and proT3N3 (61.2) subsets were similar (Table S2). Therefore, we propose that proT1N0 should be defined as stage I in the proposed classification (proStage I); the proStage II should include proT2N0, proT1N1, proT2N1, and proT3N0; proStage III should include proT3N1, proT1N2, proT2N2, and proT3N2; and proStage IVa should include proT1N3, proT2N3, and proT3N3 (Figure 2). The proposed staging system resulted in a more even and orderly increase in the HRs for DFS and OS between each stage: for DFS, the adjusted HRs [adjusted by age (>50 years vs ≤50), gender (female vs male), and chemotherapy (yes vs no)] were 1.000 for proStage I, 5.795 for proStage II, 12.705 for proStage III, and 20.438 for proStage IVa; for OS, the adjusted HRs were 1.000 for proStage I, 8.135 for proStage II, 22.069 for proStage III, and 43.059 for proStage IVa (Table 3). Furthermore, compared to the 8th edition, the proposed staging system was simpler and likely to be easier to memorize (Table S3).

**Table 3:** Risks of different overall stage for OS and DFS based on the 8th edition and proposed staging system for NPC.

Category	Hazard ratio (95% CI) for OS		Hazard ratio (95% CI) for DFS	
	8th edition	Proposed	8th edition	Proposed
Overall stage				
I	1	1	1	1
II	7.765 (1.044‐57.739)	8.135 (1.112‐59.487)	5.395 (1.668‐17.452)	5.795 (1.817‐18.483)
III	11.459 (1.560‐84.166)	22.069 (2.999‐162.381)	7.559 (2.355‐24.261)	12.705 (3.941‐40.959)
IV	30.403 (4.131‐223.768)	43.059 (5.827‐318.167)	15.527 (4.813‐50.089)	20.438 (6.291‐66.399)
c‐index	0.696 (0.666‐0.726)	0.720 (0.691‐0.749)	0.639 (0.612‐0.666)	0.661 (0.635‐0.687)

Note

Hazard ratios were calculated using an adjusted Cox proportionalhazards model. The following known important prognostic variables were included in the Cox proportional hazards model: age (>50 years vs ≤50), gender (female vs male), and chemotherapy (yes vs no). NPC: nasopharyngeal carcinoma. OS: overall survival. DFS: disease‐free survival. CI: confidence interval. c‐index: concordance index.

**Figure 3:**
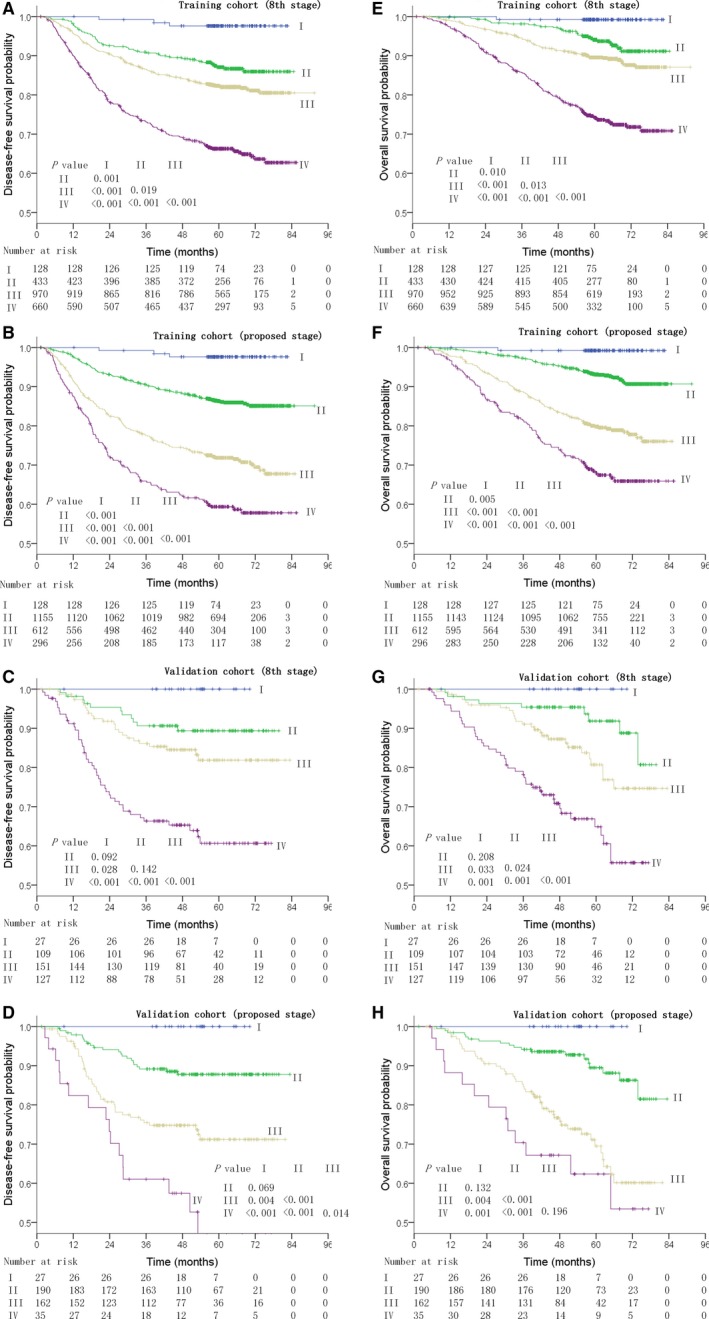
Disease‐free survival (A, C; B, D; respectively) and overall survival (E, G; F, H; respectively) for different stage groups of patients with nasopharyngeal cancer as defined by the 8th edition of the AJCC staging system, and the proposed staging system.

Using the proposed staging system, the DFS survival curves between stage II and stage III were better separated than those of the 8th edition both in the training cohort and validation cohort (Figure 3).

## 4 DISCUSSION

The TNM staging system is a global scale used to reflect the extent of disease, and is used to predict outcomes, guide treatment and facilitate the exchange of information between oncology centers worldwide. In this study, we observed a lack of separation in both LRFS, DFS, and OS between patients with the 8th edition T2 and T3 NPC. We found it reasonable that the T category classification should contain three subgroups instead of four, by merging T2 and T3. The proposed staging system is simpler than the 8th edition AJCC staging system, and furthermore, provided better distinction of hazards between adjacent stages/categories and had better prognostic value for patients with NPC in the IMRT era.

The extent of local invasion, regional lymphatic spread and distant metastasis, as reflected by the TNM staging system, are the most important prognostic factors in NPC. The TNM staging system is continually being modified to account for new developments in diagnostic and therapeutic techniques.^11^ Compared to conventional techniques, IMRT provides improved tumor target coverage with significantly better sparing of sensitive normal tissue structures during the treatment of locally advanced NPC.^12^ As a result of improved delivery efficiency, IMRT represents the optimal treatment for all stages of NPC. Moreover, the application of MRI for diagnosis and assessment of treatment response have also improved tumor control by increasing the accuracy of tumor delineation. Improved visualization of the extent of the tumor enables the radiation dose to be delivered more precisely to the GTV, and MRI staging has been confirmed to significantly improve local tumor control and survival. Furthermore, increased use of chemotherapy in patients with advanced disease has also contributed to improved local control.^13^

In the 2D‐CRT era, patients with orbit, cranial nerve, intracranial, or medial and lateral pterygoid muscle involvement have poor prognoses, and were classified as T4 disease according to the 7th AJCC staging system.^14^, ^15^ However, only orbit involvement remains a significant prognostic factor for local failure in the IMRT era.^6^ Pan et al conducted a retrospective study of 1609 patients with NPC who were staged using MRI and observed no significant differences in OS among those with infiltration of adjacent soft tissues, including the medial and lateral pterygoid muscles, prevertebral muscles and parapharyngeal space.^16^ The improved coverage of the parapharyngeal space and skull base provided by IMRT avoids the problem of low‐radiation doses to these regions (which commonly existed in the conventional field arrangement of regular 2D‐RT), leading to improved regional control in T2 and T3 NPC.^17^, ^18^ In this study, we did not identify any significant prognostic factors for LRFS in patients with 8th edition T2‐3 disease in either univariate or multivariate analysis. Therefore, simplification of the T category classification is necessary.

Medial and lateral pterygoid muscle involvement was down‐staged from T4 to T2 in the 8th AJCC staging system. These changes provide better distinction of hazards between adjacent stages/categories with respect to DFS and OS. However, the current 8th edition is not completely satisfactory. This study demonstrates the 8th edition results in a lack of separation of LRFS, DFS, and OS between stage T2 and T3 disease, and the HRs for disease failure for T2 and T3 are very similar. Furthermore, Pan et al, reported that LRFS was not significantly different between the 8th edition T2 and T3 among patients treated using IMRT at two centers (Hong Kong and mainland China; *P = *0.60).^16^ Therefore, two large cohort studies in the NPC‐epidemic area indicate that merging of T2 and T3 into T2 seems a reasonable alteration. Merging of T2 and T3 to T2 resulted in significant differences in LRFS, DFS, and OS between each T category of the proposed modification, providing improved prognostication compared to the 8th edition.

Generally, advanced T category is associated with poor local control and OS; advanced N category is associated with increased risk of distant failure and poorer OS. Lai et al found the improved treatment outcomes for patients treated with IMRT compared to 2D‐CRT were primarily due to higher local tumor control, and also demonstrated distant metastasis is now the predominant mode of treatment failure in NPC.^6^, ^19^ Hen et al reported that T category had no predictive value for local control and OS, whereas N category was a significant prognostic factor for OS.^19^ Therefore, the prognostic value of T category may have become weaker than that of N category due to the excellent local control rates. It is reasonable that the current staging system should be altered by merging the four T categories into three subsets, and we propose proT3N0‐2 (T4N0‐2 disease in the 8th edition) should be down‐staged to stage III, and N3 defined as stage IV irrespective of T category. The even and orderly increase in risk observed as tumor extent and nodal involvement increase in the proposed system support these modifications. Compared to the 8th edition, the proposed staging system has superior prognostic value, as indicated by the c‐index values. Furthermore, the proposed staging system was simpler and would be easier to memorize.

However, the proposed staging system only incorporates parameters describing the anatomic extent of the tumor, as determined by clinical and pathologic assessments. The AJCC has increasingly recognized the growing demand for more accurate and probabilistic individualized outcome predictions to develop a precision medicine approach that incorporates additional anatomic and nonanatomic prognostic factors beyond the TNM system. Therefore, additional relevant prognostic factors, such as Epstein‐Barr virus DNA load, should be considered and combined with the TNM staging system in future revisions.

This study has some limitations. First, the number of cases in the proposed stage IV subgroup was small, which might lead to the fact that after 4 years of follow‐up, the separation between the proposed stage III and IV in both the OS and DFS curves was not as wide as that between the 8th edition stage III and IV (Figure 3). Second, this was a retrospective study, which may lead to potential bias. Thus, in order to confirm our study conclusion, future prospective research is required to validate our findings. Third, the sample of the validation cohort may not be enough; hence future studies should enroll more patients. Last, the cases were from NPC‐endemic areas, so whether the conclusions from this study also apply to the nonendemic areas requires further investigation.

In conclusion, local control in NPC has improved in the modern era, and distant failure is now the main cause of disease failure. We recommended T category classification should contain three subgroups instead of four by merging T2 and T3 into T2, and proT3N0‐2 (T4N0‐2 in the 8th edition) should be down‐staged to stage III in future versions of the AJCC staging system for NPC. This proposed staging system provides better distinction of hazards between adjacent stages/categories and has superior prognostic value for patients with NPC in the IMRT era.
