# Supplementary material for: The development and external validation of simplified T category classification for nasopharyngeal carcinoma to improve the prognostic value in the intensity‐modulated radiotherapy era
Source: Cancer Med. 2019 Apr 4;8(5):2213–22. doi: 10.1002/cam4.2131 (PMC6536995; doi:10.1002/cam4.2131)
Supplement: Supplementary file 1 [file CAM4-8-2213-s001.docx]

| prognostic factors | 5y-LRFS  (Yes vs. No.) | HR* | 95% CI | *P** |
| --- | --- | --- | --- | --- |
| Parapharyngeal space | 93.5% vs.95.5% | 1.500 | 0.865-2.601 | 0.149 |
| medial pterygoid | 92.9% vs.94.4% | 1.398 | 0.816-2.396 | 0.223 |
| lateral pterygoid | 90.8% vs 94.3% | 1.613 | 0.509-5.117 | 0.417 |
| prevertebral muscles | 94.3% vs 94.1% | 1.047 | 0.662-1.654 | 0.846 |
| skull base | 94.1% vs.94.4% | 1.076 | 0.641-1.807 | 0.781 |
| cervical vertebra | 100% vs.94.2% | 0.05 | - | 0.648 |
| paranasal sinuses | 90.6% vs.94.5% | 1.763 | 0.907-3.426 | 0.095 |

**Supplementary Table S1.** Summary of univariate analysis of prognostic factors for local relapse-free survival in T2-3 nasopharyngeal carcinoma.

Multivariate analyses were performed using the Cox proportional hazards model to test for independent significance by backward elimination of insignificant explanatory variables. Since the factors above were all insignficant, no multivariate analyses results were shown. LRFS: local relapse-free survival. HR: hazard ratio. CI: confidence interval.

**Supplementary Table S2.** Risks of proposed T and N categories for OS and DFS

| **Subgroups**  **(no. of patients)** | **HR (95% CI)** | | |  |
| --- | --- | --- | --- | --- |
|  | **OS** | **DFS** | |  |
| proT1N0 (128) | 1 | | 1 | |
| proT1N1 (190) | 6.092 (0.772-48.099) | | 3.831 (1.123-13.073) | |
| proT2N0 (180) | 7.583 (0.986-58.320) | | 3.780 (1.113-12.833) | |
| proT2N1 (785) | 8.949 (1.238-64.669) | | 5.958 (1.892-18.764) | |
| proT3N0 (45) | 15.089 (1.816-125.396) | | 7.022 (1.862-26.482) | |
| proT1N2 (254) | 19.844 (2.316-170.066) | | 10.967 (2.907-41.369) | |
| proT2N2 (65) | 22.161 (3.038-161.639) | | 11.575 (3.617-37.042) | |
| proT3N1 (32) | 23.550 (3.248-170.723) | | 11.433 (3.592-36.387) | |
| proT3N2 (216) | 27.420 (3.564-210.934) | | 13.311 (3.877-45.696) | |
| proT2N3 (39) | 38.277 (5.282-277.397) | | 15.284 (4.786-48.809) | |
| proT1N3 (206) | 43.200 (5.520-338.071) | | 17.830 (5.076-62.628) | |
| proT3N3 (51) | 61.203 (8.248-454.147) | | 26.359 (7.976-87.113) | |

Hazard ratios were calculated using an adjusted Cox proportional-hazards model. The following known important prognostic variables were included in the Cox proportional hazards model: age (> 50 years vs. ≤50), gender (female vs. male), and chemotherapy (yes vs. no). no.: number. HR: hazard ratio. CI: confidence interval. OS: overall survival. DFS: disease-free survival.

**Supplementary Table S3.** Classification criteria and stage grouping according to the 8^th^ edition of the AJCC and proposed staging system.

|  | **The 8th edition** | | | | | | **Proposed staging system** | | | | | |
| --- | --- | --- | --- | --- | --- | --- | --- | --- | --- | --- | --- | --- |
| T catogeries | | | |  | | |  |  |  |  |  |  |
|  | T1: Nasopharynx, oropharynx or nasal cavity without parapharyngeal extension | | | | | | T1: Nasopharynx, oropharynx or nasal cavity without parapharyngeal extension | | | | | |
|  | T2: Parapharyngeal extension, adjacent soft tissue involvement (medial pterygoid, lateral pterygoid, prevertebral muscles) | | | | | | T2: Parapharyngeal extension, adjacent soft tissue involvement (medial pterygoid, lateral pterygoid, prevertebral muscles) and Bony structures (skull base, cervical vertebra) and/or paranasal sinuses | | | | | |
|  | T3: Bony structures (skull base, cervical vertebra) and/or paranasal sinuses | | | | | |  | | | | | |
|  | T4: Intracranial extension, cranial nerve, hypopharynx, orbit, extensive soft tissue involvement (beyond the lateral surface of the lateral pterygoid muscle, parotid gland) | | | | | | T3: Intracranial extension, cranial nerve, hypopharynx, orbit, extensive soft tissue involvement (beyond the lateral surface of the lateral pterygoid muscle, parotid gland) | | | | | |
| N catogeries | | | |  | | |  |  |  |  |  |  |
|  | N0: No regional lymph node metastasis | | | | | | N0: No regional lymph node metastasis | | | | | |
|  | N1: Retropharyngeal (regardless of laterality) Cervical: unilateral, ≤6 cm, and above caudal border of cricoid cartilage | | | | | | N1: Retropharyngeal (regardless of laterality) Cervical: unilateral, ≤6 cm, and above caudal border of cricoid cartilage | | | | | |
|  | N2: Cervical: bilateral, ≤6 cm, and above caudal border of cricoid cartilage | | | | | | N2: Cervical: bilateral, ≤6 cm, and above caudal border of cricoid cartilage | | | | | |
|  | N3: >6 cm and/or below caudal border of cricoid cartilage (regardless of laterality) | | | | | | N3: >6 cm and/or below caudal border of cricoid cartilage (regardless of laterality) | | | | | |
| **Stage group** | | | |  | | |  |  |  |  |  |  |
|  |  | T1 | T2 | T3 | T4 |  | T1 | T2 | T3 |  |  |  |
|  | N0 | I | II | III | IVa |  | I | II | III |  |  |  |
|  | N1 | II | II | III | IVa |  | II | II | III |  |  |  |
|  | N2 | III | III | III | IVa |  | III | III | III |  |  |  |
|  | N3 | IVa | IVa | IVa | IVa |  | IVa | IVa | IVa |  |  |  |

Abbreviations: AJCC, American Joint Committee on Cancer.
